# Supplementary material for: New cycle, same old mistakes? Overlapping vs. discrete generations in long-term recurrent selection
Source: BMC Genomics. 2022 Oct 31;23:736. doi: 10.1186/s12864-022-08929-3 (PMC9624058; doi:10.1186/s12864-022-08929-3)
Supplement: Supplementary file 14 — Supplementary Material 14 [file 12864_2022_8929_MOESM14_ESM.docx]

**Table S6.** For the RS-AY scenarios, results of one-sample *t* tests of mean parental age at year 40 for each overlapped scenario compared to μ = 3.67. Means were compared to 3.67 because it was the average generation interval in years for the RS-AY scenario under discrete selection. The Bonferroni-corrected α value was 0.025.

| **Scenario** | | ***t*** | | | **d.f.** | ***P*** |
| --- | --- | --- | --- | --- | --- | --- |
| Overlapping Phenotypic | | 8.071 | | | 9 | **< 0.0001** |
| Overlapping Genomic | | 12.451 | | | 9 | **< 0.0001** |
|  |  | |  |  |  |  |

**Table S7.** For the RS-AY scenarios, results of one-sample *t* tests of mean year error bias at year 40 for each overlapped scenario compared to μ = 1. Means were compared to 1 because year error bias is by definition 1 under discrete selection. The Bonferroni-corrected α value was 0.025.

| **Scenario** | ***t*** | **d.f.** | ***P*** |
| --- | --- | --- | --- |
| Overlapping Phenotypic | 3.589 | 9 | **0.006** |
| Overlapping Genomic | 0.919 | 9 | 0.382 |
