# Supplementary material for: New cycle, same old mistakes? Overlapping vs. discrete generations in long-term recurrent selection
Source: BMC Genomics. 2022 Oct 31;23:736. doi: 10.1186/s12864-022-08929-3 (PMC9624058; doi:10.1186/s12864-022-08929-3)
Supplement: Supplementary file 15 — Supplementary Material 15 [file 12864_2022_8929_MOESM15_ESM.docx]

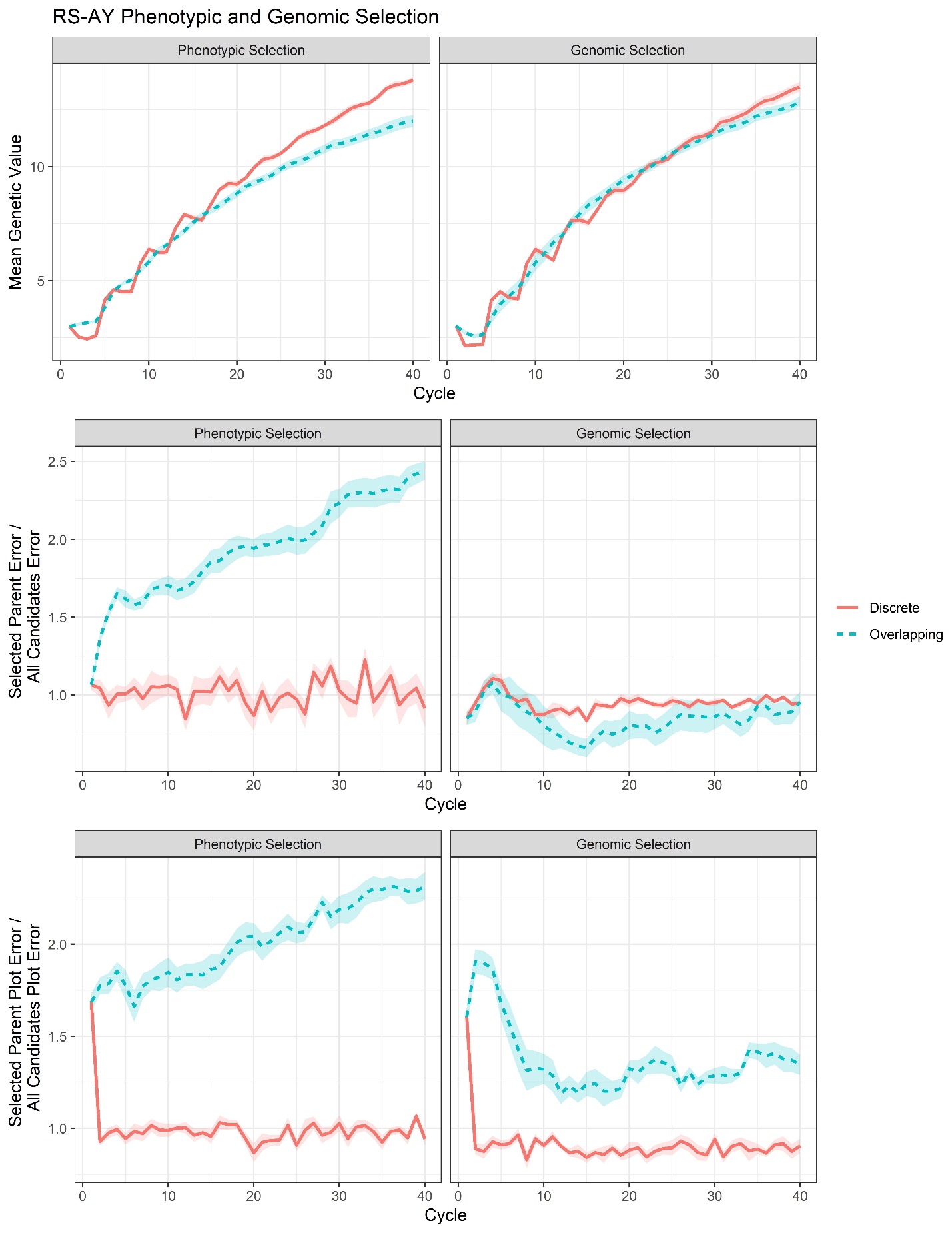


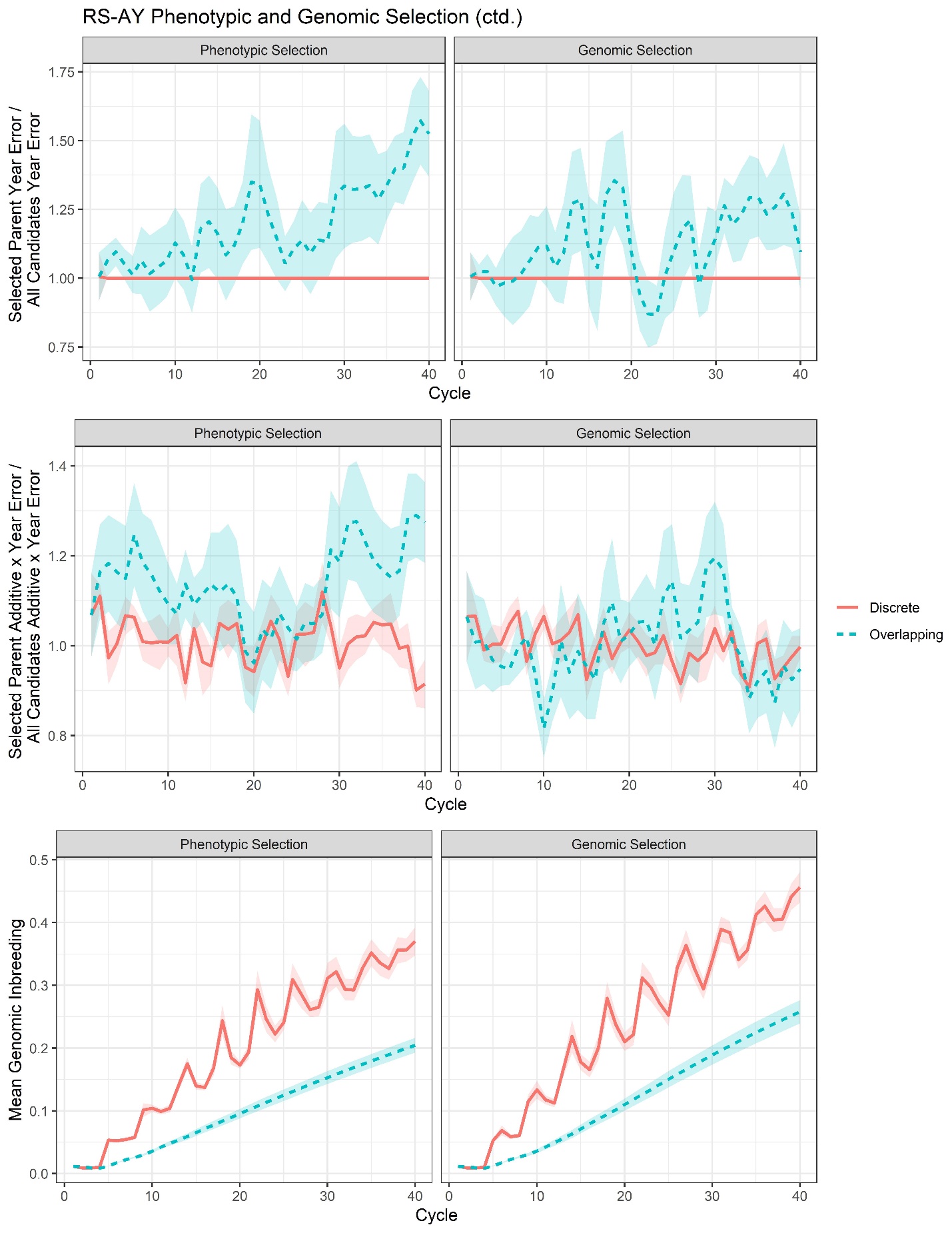


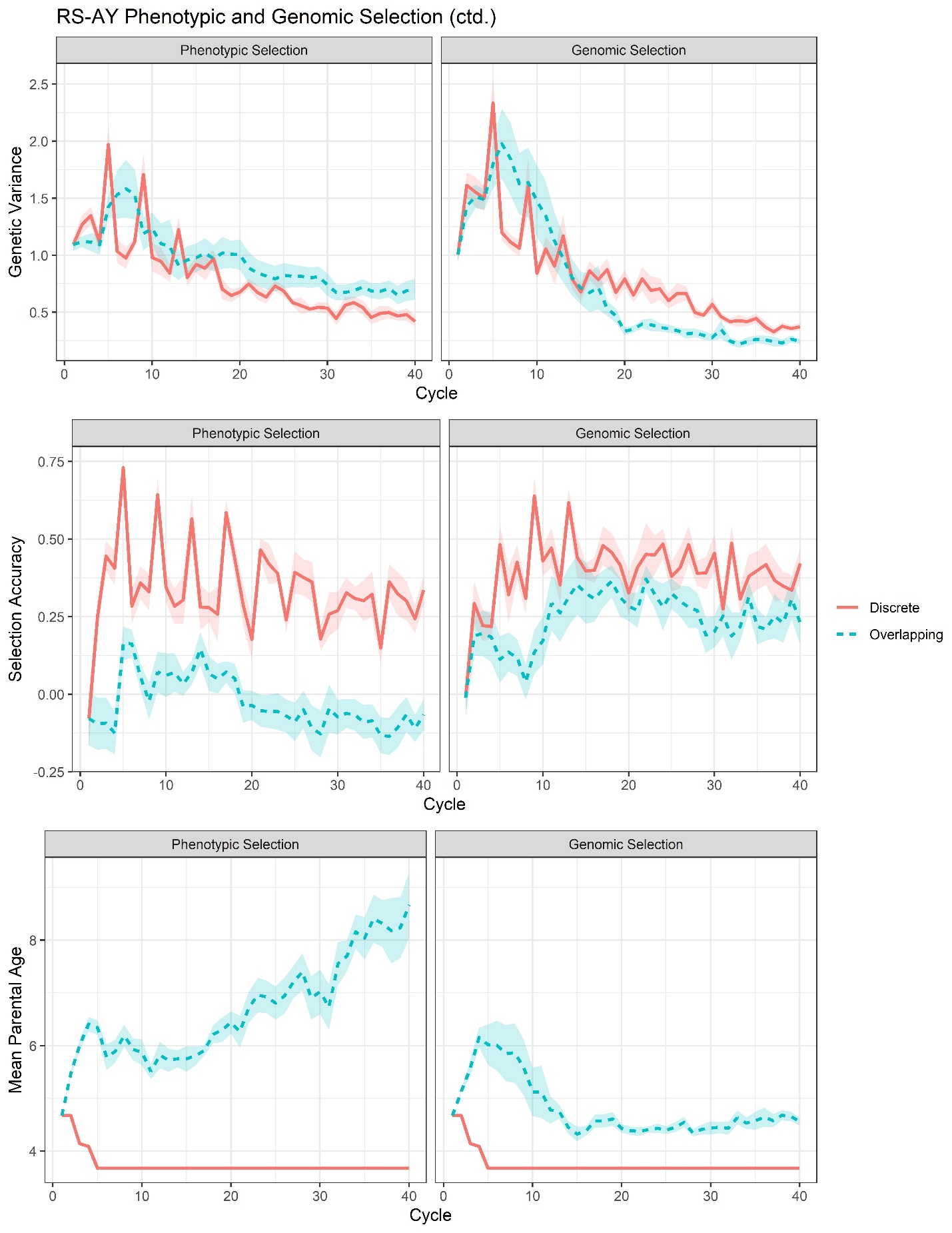


**Supplemental File 26, Figure S14.** Plots of all responses recorded for the RS-AY scenarios, including both phenotypic and genomic selection.
