# Supplementary material for: New cycle, same old mistakes? Overlapping vs. discrete generations in long-term recurrent selection
Source: BMC Genomics. 2022 Oct 31;23:736. doi: 10.1186/s12864-022-08929-3 (PMC9624058; doi:10.1186/s12864-022-08929-3)
Supplement: Supplementary file 22 — Supplementary Material 22 [file 12864_2022_8929_MOESM22_ESM.docx]

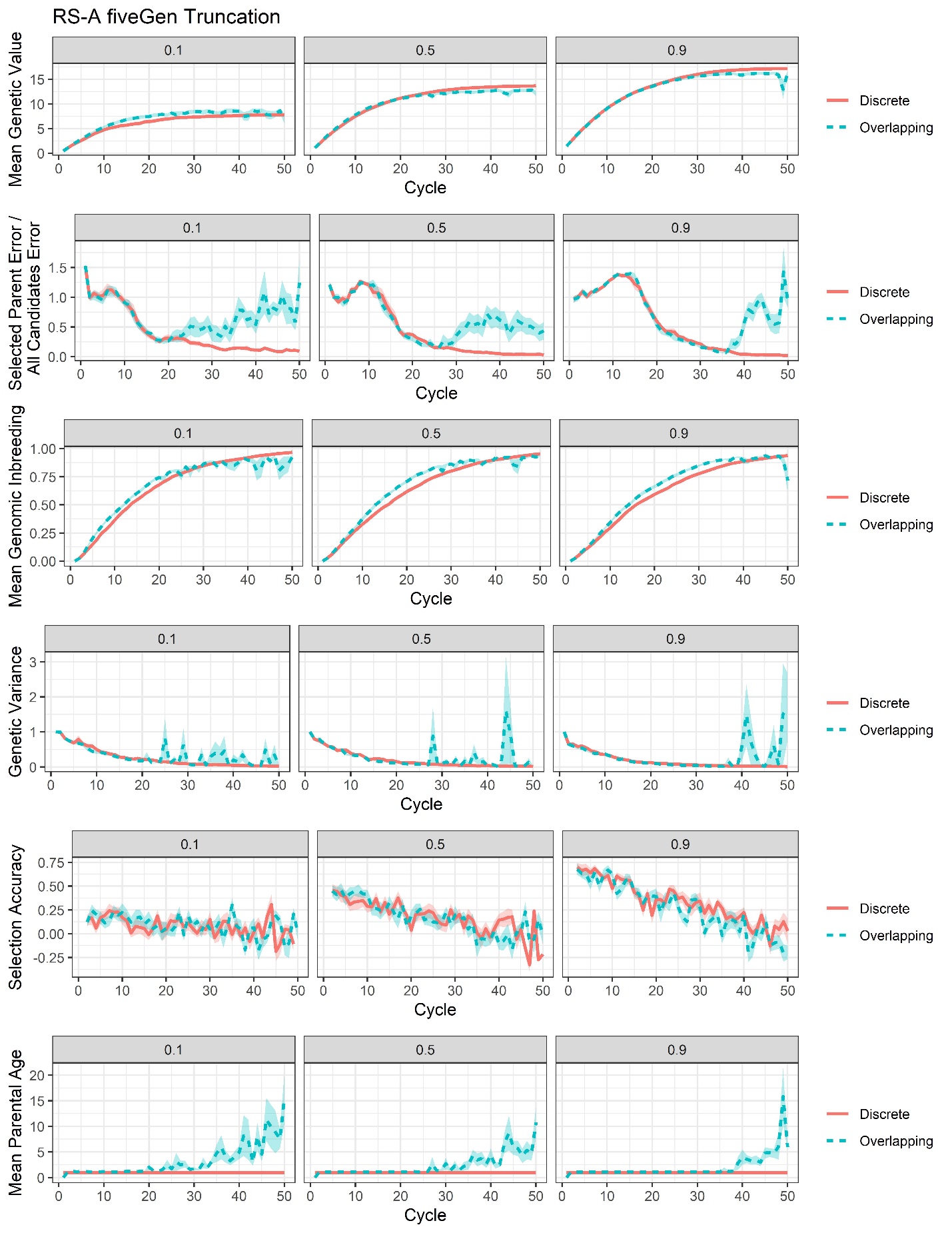


**Supplemental File 19, Figure S7.** Plots of all responses recorded for the RS-A genomic truncation selection with training on the previous five generations scenario (fiveGen Trunc).
