# Supplementary material for: New cycle, same old mistakes? Overlapping vs. discrete generations in long-term recurrent selection
Source: BMC Genomics. 2022 Oct 31;23:736. doi: 10.1186/s12864-022-08929-3 (PMC9624058; doi:10.1186/s12864-022-08929-3)
Supplement: Supplementary file 25 — Supplementary Material 25 [file 12864_2022_8929_MOESM25_ESM.docx]

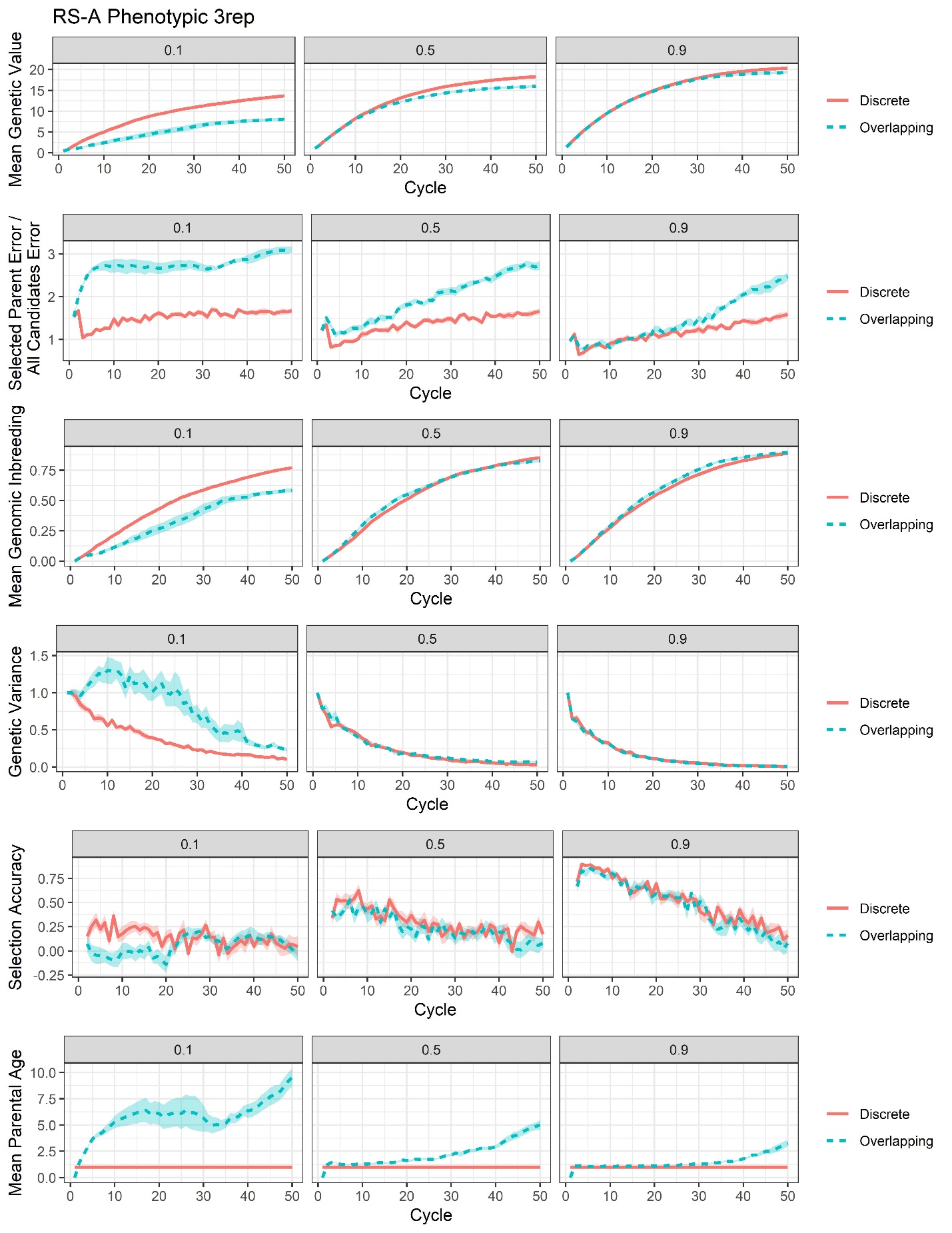


**Supplementary File 16, Figure S4.** Plots of all responses recorded for the RS-A phenotypic 3rep selection scenario.
