# Supplementary material for: New cycle, same old mistakes? Overlapping vs. discrete generations in long-term recurrent selection
Source: BMC Genomics. 2022 Oct 31;23:736. doi: 10.1186/s12864-022-08929-3 (PMC9624058; doi:10.1186/s12864-022-08929-3)
Supplement: Supplementary file 28 — Supplementary Material 28 [file 12864_2022_8929_MOESM28_ESM.docx]

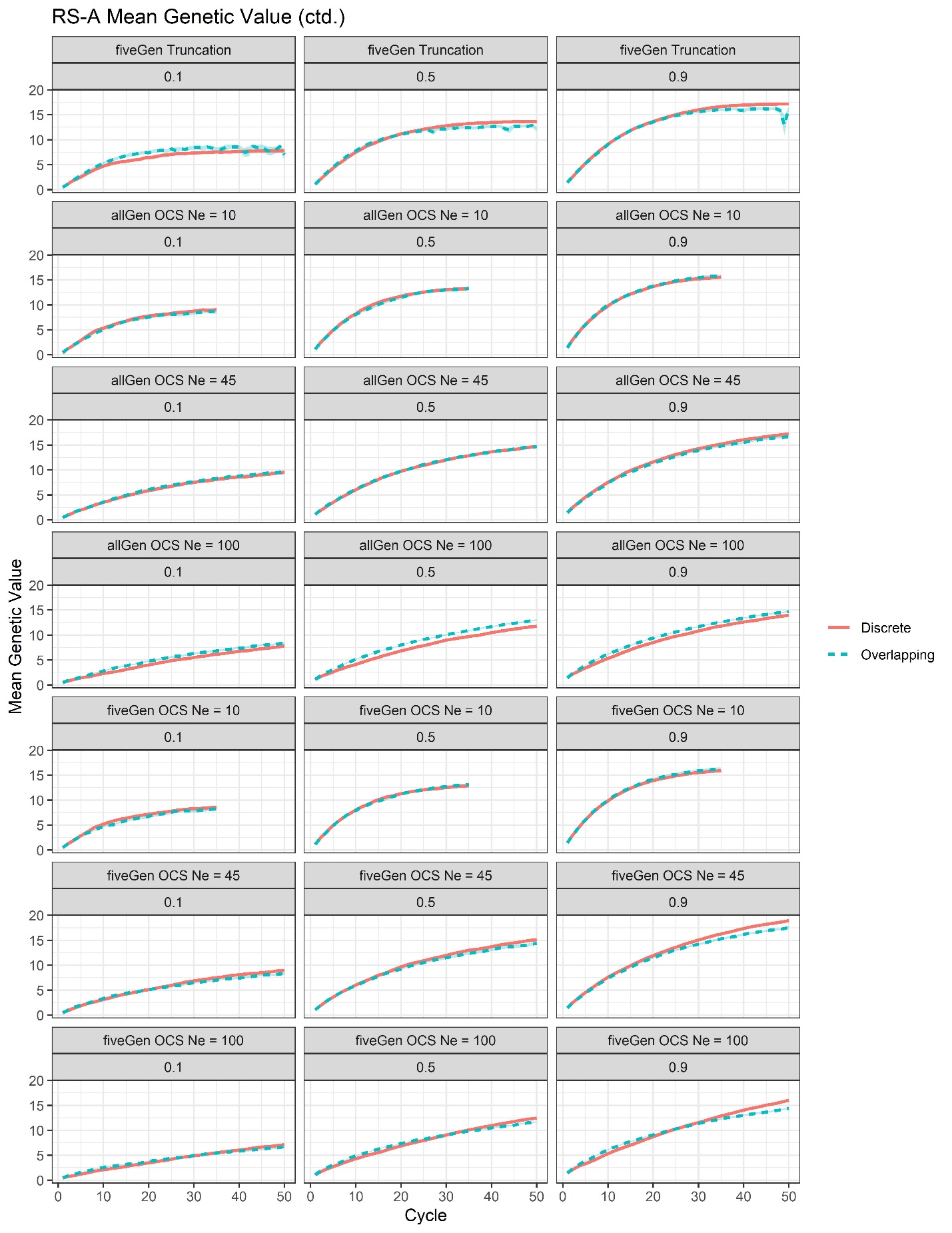


**Supplemental File 13, Figure S1.** Plots of mean genetic value by cycle surrounded by 95% confidence intervals for the RS-A scenarios with genomic truncation selection and training on the previous five generations (fiveGen Trunc) as well as all RS-A OCS scenarios.
